# Medical Oncology or Surgical Oncology: Which Branch Should Be Started in Esophageal Cancer Diagnostic Evaluation?

**DOI:** 10.7759/cureus.22286

**Published:** 2022-02-16

**Authors:** Özgen Ahmet Yıldırım, Erkan Erdur

**Affiliations:** 1 Department of Medical Oncology, University of Health Sciences Ankara Dr Abdurrahman Yurtarslan Oncology Training and Research Hospital, Ankara, TUR; 2 Division of Medical Oncology, Department of Internal Medicine, Gazi Yasargil Training and Research Hospital, Diyarbakir, TUR

**Keywords:** neoadjuvant chemotherapy, esophageal cancer, prognostic risk factors, multimodal treatment, treatment delays

## Abstract

Objective

We evaluated the effect of the branch of the cancer specialist (medical oncologist versus surgical oncologist) who initially examines a patient on treatment delay. The objective was to evaluate whether surgical oncology and medical oncology clinics, which have different operating styles, impact the timeliness of treatment. Additionally, we investigated the prognostic impact of the clinical and treatment-related factors in patients with esophageal cancer treated at our center.

Methods

This was a retrospective single-center study. The prognostic impact of resection type (R0 or R1-2), multimodal treatment, lymphovascular invasion (LVI), perineural invasion (PNI), lymph node metastases, cachexia at the time of diagnosis, smoking, and diagnostic application of endoscopic ultrasound was evaluated. Patients were stratified according to whether the orientation and management processes were based on a multimodal approach and whether they were first examined by a surgical oncologist or a medical oncologist for diagnostic workup and management. The impact of the management approach on progression-free survival (PFS) was evaluated.

Results

Use of a multimodal approach in patients with esophageal cancer was associated with longer PFS (26.7 vs 13.9 months, p = 0.002). LVI and cachexia were associated with shorter PFS (16.1 vs 29.4 months, p = 0.044 and 14.6 vs 29.0, p = 0.019, respectively). The first appointment of the patients in the medical oncology department was associated with shorter treatment delay (54 [IQR: 36-71] vs 31 [IQR: 24-48] days, p < 0.001).

Conclusions

Our findings suggest that the first appointment of patients in the medical oncology department may lead to a more systematic workup and treatment progress. We believe that systematic use of multimodal approaches for esophageal cancer may confer prognostic benefits.

## Introduction

Esophageal cancer is the eighth most common type of cancer and the sixth most common cause of cancer-related deaths in the world. Owing to the lack of serosal layer and its anatomical proximity to the vital organs, esophageal cancer is often diagnosed at an advanced stage. Approximately 75% patients are not amenable to curative treatment owing to the advanced disease stage. The reported five-year survival rate of these patients is 15-20%. Ethnicity, genetic factors, and lifestyle-related factors play an important role in the pathogenesis of esophageal cancer [1]. Although the incidence of adenocarcinoma has increased in recent years, squamous cell carcinoma is still the most common type. There are etiological differences between the two types. Obesity, gastroesophageal reflux, and smoking play a more important role in the etiology of adenocarcinoma. Alcohol intake, smoking, and local dietary habits are more commonly associated with squamous cell cancer [2].

Esophageal cancer can be asymptomatic in the early stages. Patients may develop swallowing difficulties and impaired peristalsis. In the advanced stage, patients typically show weight loss, progressive dysphagia, and odynophagia [3]. Adenocarcinoma typically occurs at the distal end of the esophagus. Squamous cell carcinoma more commonly occurs in the middle part of the esophagus, but adenocarcinoma can also be seen [4].

Computed tomography (CT) and positron emission tomography (PET) are useful diagnostic imaging modalities. Local invasion status can be evaluated using endoscopic ultrasonography (EUS) [5]. Surgical resection is the primary treatment for resectable localized tumors. Use of perioperative chemotherapy and chemoradiotherapy in addition to surgery confers a survival benefit in patients with locally advanced tumors. Neoadjuvant chemoradiotherapy has been shown to be superior to neoadjuvant chemotherapy in patients with squamous cell carcinoma. In patients with adenocarcinoma, use of neoadjuvant chemotherapy alone also confers a strong survival benefit [6].

Studies have shown the benefit of adjuvant chemotherapy in patients with lymph node metastasis who have undergone preoperative chemoradiotherapy and have been operated [7].

The ongoing COVID-19 pandemic has had an adverse impact on the management of cancer patients, often leading to treatment delays. Various studies have assessed the prognostic implications of treatment delay. In a study evaluating esophagectomy cases, the adverse prognostic impact of treatment delay was observed both in the group receiving neoadjuvant therapy and in cases that were operated directly under pandemic conditions [8]. According to a study, there was a significant decrease in treatment delay in patients with gastroesophageal cancers over the last 10 years, and this phenomenon contributed to improved prognosis of these patients [9]. A literature review by DE Rosa et al. revealed that the diagnostic and therapeutic process of gastrointestinal malignancies in the conditions of the COVID-19 pandemic can be accomplished with acceptable delays without prognostic worsening [10].

In this study, we evaluated the effect of the branch of the cancer specialist (medical oncologist versus surgical oncologist) who first examined the patient on treatment delay. The objective was to evaluate whether surgical oncology and medical oncology clinics, which have different operating styles, make a difference in this respect. Additionally, we investigated the prognostic impact of the clinical and treatment-related factors in patients with esophageal cancer treated at our center.

## Materials and methods

We retrospectively evaluated the data of patients diagnosed with esophageal cancer who were followed up at the Diyarbakır Gazi Yaşargil Training and Research Hospital between January 2011 and August 2021. Medical records of 141 patients were reviewed, and 64 patients were included in the study. Patients with gastroesophageal junction tumors were not included. Cases evaluated as esophageal distal end cancer in the endoscopy report were included.

Data pertaining to the following demographic and diagnostic parameters were evaluated: age, sex, family history, smoking and alcohol use, initial Eastern Cooperative Oncology Group (ECOG) performance score, comorbidities, tumor location, clinical stage, pathological stage, histopathological type, grade, lymphovascular invasion (LVI), perineural invasion (PNI), metastatic lymph node status, PET-CT status, and EUS application status.

Treatment-related parameters were the presence of surgical treatment, resection result (R0 or R1-2), and information related to chemotherapy and radiotherapy. In addition, patients were divided into two groups: those who received multimodal treatment and those who did not. Definitive radiotherapy lower limit was accepted as 50 Gy.

In terms of factors affecting progression-free survival (PFS), resection type (R0 or R1-2), receiving or not receiving multimodal treatment, presence of LVI, PNI, presence of lymph node metastases, presence of cachexia at the time of diagnosis, smoking history of >30 packs/year, and application of EUS for diagnosis were evaluated.

In addition, patients were stratified depending on the orientation and management processes of the patients to the multimodal approach, i.e., whether they were first examined by a surgical oncologist or a medical oncologist during the diagnostic process. The effect of this situation on PFS was evaluated.

SPSS Version 26.0 (IBM Corp., Armonk, NY, United States) was used for data analysis. The normality of distribution of variables was evaluated using the Shapiro-Wilk Francia test, while homogeneity of variance was evaluated using the Levene test. For comparison of normally distributed variables between two independent groups, the independent samples t-test was used together with the Bootstrap results, while the Mann-Whitney U test was used for non-normally distributed continuous variables together with the Monte Carlo results. For comparison of categorical variables, the Fisher exact test and Fisher-Freeman-Halton test were used along with the Monte Carlo simulation technique. Quantitative variables are presented as mean ± standard deviation or as median (percentile 25%/percentile 75%), while categorical variables are shown as frequency (%). Variables were analyzed at 95% confidence level, and p-values of less than 0.05 were considered indicative of statistical significance.

The study was approved by the Ethics Committee of the Health Sciences University Diyarbakir Gazi Yaşargil Training and Research Hospital (No: 896, dated: 08.10.2021). Ethics approval was applied in accordance with the Declaration of Helsinki.

## Results

The majority of patients in our cohort were male (69%, n = 44). The median age of patients was 57 years (range: 26-74 years). The percentage of smokers and those who used alcohol was 63% (n = 40) and 14% (n = 9), respectively. Of the patients, 44% (n = 28) had hypertension, 11% (n = 7) had diabetes, and 5% (n = 3) had cardiac disease. The distribution of patients with upper esophageal tumors, middle esophagus tumors, and lower esophagus tumors was 9%, 39%, and 52%, respectively. The ECOG score was 0 in 11%, 1 in 80%, and 2 in 9% patients. The histopathological diagnosis was squamous cell carcinoma in 81% patients and adenocarcinoma in 19% patients; 11% cases were well differentiated, 61% were moderately differentiated, and 28% were poorly differentiated or with signet cell histology. LVI was seen in 23% and PNI in 16%. Pathologically defined lymph nodes over 1 cm were observed radiologically in 25% cases. PET-CT was used for diagnosis in all patients. EUS was used for staging in 17% cases. Overall, 9% of the patients had stage 2 disease, 77% had stage 3 disease, and 14% had stage 4 disease. Clean surgical margin resection was achieved in 94% of patients who underwent surgery. Neoadjuvant chemotherapy was administered to 12.5% patients. Neoadjuvant chemoradiotherapy was administered to 45% patients. Definitive chemoradiotherapy was applied to 22% patients. Upfront surgery was performed in 12.5% cases. Multimodal treatment approach was applied in 45% patients. With respect to the clinical response to neoadjuvant treatments, we found that 50% of the cases achieved clinical complete response. However, the rate of pathological complete response was 25%. The demographic, diagnostic, and treatment characteristics are summarized in Table 1.

**Table 1:** Demographic, diagnostic, and treatment characteristics of patients **Irresponsive or minimal response ECOG, Eastern Cooperative Oncology Group performance score; PET CT, positron emulsion tomography computerized tomography; EUS, endoscopic ultrasonography; CR, complete remission; PR, partial regression; Irr, irresponsive

Patient characteristics (n=64)	
Age, median (min-max)	57 (26-74)
Gender, n (%)	
Female	20 (31%)
Male	44 (69%)
Tobacco n (%)	40 (63%)
Alcohol	9 (14%)
Comorbidities n (%)	
Hypertension	28 (44%)
Diabetes	7 (11%)
Cardiac disease	3 (5%)
Anatomic localization n (%)	
Upper	6 (9%)
Middle	25 (39%)
Low end	33 (52%)
ECOG score n (%)	
0	7 (%11)
1	51 (80%)
2	6 (9%)
Histologic type n (%)	
Squamous	52 (81%)
Adenocarcinoma	12 (19%)
Differentiation, n (%)	
Well	7 (%11)
Moderate	39 (61%)
Poor or signet cell	18 (28%)
Lymphovascular invasion n (%)	15 (23%)
Perineural invasion n (%)	10 (16%)
Positive lymph node in imaging, n (%)	16 (25%)
Use of PET CT in diagnosis n (%)	64 (100%)
Use of EUS in diagnosis n (%)	11 (17%)
Clinical stage n (%)	
2	6 (9%)
3	49 (77%)
4	9 (14%)
Surgery result n (%)	
R0	47 (94%)
R1	3 (6%)
Neoadjuvant chemotherapy n (%)	8 (12.5%)
Neoadjuvan chemoradiotherapy n (%)	29 (45%)
Definitive chemoradiotherapy n (%)	14 (22%)
Up-front surgery n (%)	8 (12.5%)
Multimodal treatment n (%)	29 (45%)
Neoadjuvant treatment clinical response n (%)	
CR	28 (50%)
PR	25(45%)
Irr**	3 (5%)
Neoadjuvant treatment pathological response n (%)	
CR	14(25%)
PR	39 (61%)
Irr**	3 (5%)

Resection type (R0 versus R1-2) showed numerical differences in PFS. Receiving or not receiving multimodal treatment had a significant effect on PFS (p = 0.002). Patients with LVI had significantly shorter PFS compared to those without LVI (p = 0.044). Cachexia at diagnosis was associated with significantly shorter PFS (p = 0.019). The PFS of patients who underwent EUS during the diagnostic process was significantly longer (p = 0.047). The factors affecting PFS are presented in Table 2.

**Table 2:** Prognostic factors and related PFS and treatment delay Univariate analysis of factors **Cannot be applied due to the low number of patients. ***From first hospital registration to start of the treatment approach PFS, progression-free survival; LVI, lymphovascular invasion; PNI, perineural invasion; EUS, endoscopic ultrasonography

Factor	PFS (months), median (interquartile range): 20.8 (11.1-33.2)
Gender	
Male	19.3 (13.6-24.1)
Female	24.1 (14.3-32.0)
p-Value	0.114
Resection result	
R0	19.3 (12.7-25.6)
R1	11.7
p-Value	n.a**
Multimodal treatment	
Yes	26.7 (20.8-33.2)
No	13.6 (10.4-17.8)
p-Value	0.002
LVI	
Yes	16.1 (11.5-24.1)
No	29.4 (24.4-36.8)
p-Value	0.044
PNI	
Yes	19.8 (14.2-24.4)
No	26.7 (15.4-31.2)
p-Value	0.091
Cachexia at the time of diagnosis	
Yes	14.6 (10.2-20.3)
No	29.0 (24.2-36.4)
p-Value	0.019
Tobacco more than 30 pack	
Yes	20.1 (14.4-28.4)
No	22.4 (15.1-29.1)
p-Value	0.39
Evaluated with EUS	
Yes	30.1 (26.2-40.1)
No	18.7 (22.2-27)
p-Value	0.047
First appointment (of oncology)	Treatment delay (days***)
Surgery (n=29)	54 (36-71)
Medical oncology (n=35)	31 (24-48)
p-Value	<0.001

We evaluated the effect of the branch of the patient's first oncology appointment as the primary end-point. It was found that patients who started the process with medical oncology showed shorter treatment delay (54 (36-71) vs 31 (24-48) days, p < 0.001) (Table 2).

There was no significant association between comorbidities and histological type. However, all patients with adenocarcinoma had a history of hypertension. In addition, six of the seven diabetic patients were diagnosed with adenocarcinoma. There was no significant association between tumor differentiation status and PFS due to the small sample size. The mean PFS in the well-differentiated group, moderately-differentiated group, and poorly differentiated or signet ring cell group was 37.8 months, 24.1 months, and 11.9 months, respectively.

## Discussion

In this study, we evaluated the demographic, diagnostic, and treatment-related data of patients with esophageal cancer treated at a single center. The age and sex distribution in our cohort was comparable to that reported in previous studies. Moreover, the rate of smoking was high, which is consistent with previous studies [3].

Although no significant association between comorbidities and histological type was observed in our cohort, all patients with adenocarcinoma histology had a history of hypertension. In addition, six of the seven diabetic patients were diagnosed with adenocarcinoma. These findings support the relationship of metabolic syndrome and obesity with esophageal adenocarcinoma. Esophageal cancer consists of two histological subtypes. Although the incidence of adenocarcinoma has increased in recent years, squamous cell carcinoma is the most common histological type. While adenocarcinoma is most commonly seen at the distal end, the squamous cell type is found in all three regions of the esophagus [11].

To the best of our knowledge, this is the first study to evaluate the effect of the branch of the first applied oncology clinic on treatment delay. However, in our country, the diagnostic process of surgical clinics may be slower than that in medical clinics due to operation routines and other occupations. For this reason, the treatment planning process of a cancer patient in the medical clinic may be more systematic and faster, which may have improved the prognosis. In patients evaluated in the medical oncology clinic, the tendency to administer neoadjuvant treatments was higher than that in surgical clinics. All eight patients who underwent upfront surgery in our study were cases that consulted the surgery clinic. The CROSS study clearly demonstrated the survival benefit of neoadjuvant chemoradiotherapy in patients with locally advanced esophageal cancer [12].

The prognostic factors reported to affect the survival of esophageal cancer patients are age, performance score, stage, tumor grade, neoadjuvant therapy, tumor invasion depth, lymph node involvement, LVI, and surgical margin negativity [13-15]. In our study, since the survival data were not yet completed, we evaluated the PFS. We found that patients who received multimodal treatment approach showed longer PFS. The absence of inoperable cases in our cohort may have contributed to this result. Moreover, high PFS may have been observed due to the relatively early disease stage of patients who were evaluated with EUS. Presence of LVI and cachexia was found to be associated with shorter PFS, which is consistent with the literature.

Multidisciplinary approach is the current standard for the management of esophageal cancer, especially for adenocarcinoma. Allum et al. showed that the addition of neoadjuvant chemotherapy to treatment improves survival outcomes [16]. The Medical Research Council Adjuvant Gastric Infusional Chemotherapy study compared three courses of epirubicin, cisplatin, and 5-fluorouracil (ECF) treatment preoperatively and postoperatively with surgery alone. The five-year overall survival rate in the perioperative chemotherapy group was significantly greater than that in the surgery alone group (36% vs 23%, respectively; p = 0.009) [17].

The multidisciplinary approach should not be perceived only as a trimodal application of treatment. The decision-making process for unimodal versus bimodal treatment should be a collaborative effort involving surgery, medical oncology, radiation oncology, and, when necessary, radiology and nuclear medicine departments. Studies have demonstrated prognostic benefits of obtaining a multi-branch opinion when deciding on surgery for esophageal cancer, which is an operation with high morbidity and mortality [18-20]. Our study showed that the time from the first admission of these patients to the initiation of specific treatment may be prolonged enough to be of prognostic significance. In these patients, the time span from clinical suspicion, endoscopic diagnosis, pathological examination, to the subsequent multidisciplinary treatment decision-making can be prolonged, especially in relatively small centers. In this context, our study suggests that the patient's application to a medical oncologist prior to the surgeon may be of prognostic relevance.

Some limitations of our study should be acknowledged. This was a retrospective, single-center study and thus some statistical analyses could not be performed due to the limited number of patients. We believe that further indepth studies examining the branch of the first cancer physician appointments in the diagnostic process of the patients may help improve the oncology process. Moreover, further studies should also focus on the prognostic value of diagnostic approaches for early stage esophageal cancer. Another limitation of our study is that it may not be easy to generalize the finding to every center or health system. The cooperation of the branches will be good for systematic diagnostic progress in specialized centers. However, our study is to examine the dynamics that take place in distant cities rather than the hospitals of the central cities of our country. We believe that the data we obtained in terms of developing countries are meaningful.

## Conclusions

In our study, we found that the multimodal approach for the management of patients with esophageal cancer was associated with longer PFS. Consistent with the literature, LVI and cachexia were associated with shorter PFS. Our findings suggest that the first appointment of the patients in the medical oncology branch may be associated with a more systematic workup and management of the patient, potentially improving treatment outcomes. We believe that the systematic application of multimodal approaches for the management of esophageal cancer may confer prognostic benefits.
